# Supramolecular Structure of Phenyl Derivatives of Butanol Isomers

**DOI:** 10.1021/acs.jpcb.2c01269

**Published:** 2022-05-06

**Authors:** Joanna Grelska, Karolina Jurkiewicz, Andrzej Burian, Sebastian Pawlus

**Affiliations:** † A. Chełkowski Institute of Physics, University of Silesia in Katowice, ul. 75 Pułku Piechoty 1, 41-500 Chorzów, Poland; ‡ Silesian Center for Education and Interdisciplinary Research, ul. 75 Pułku Piechoty 1A, 41-500 Chorzów, Poland

## Abstract

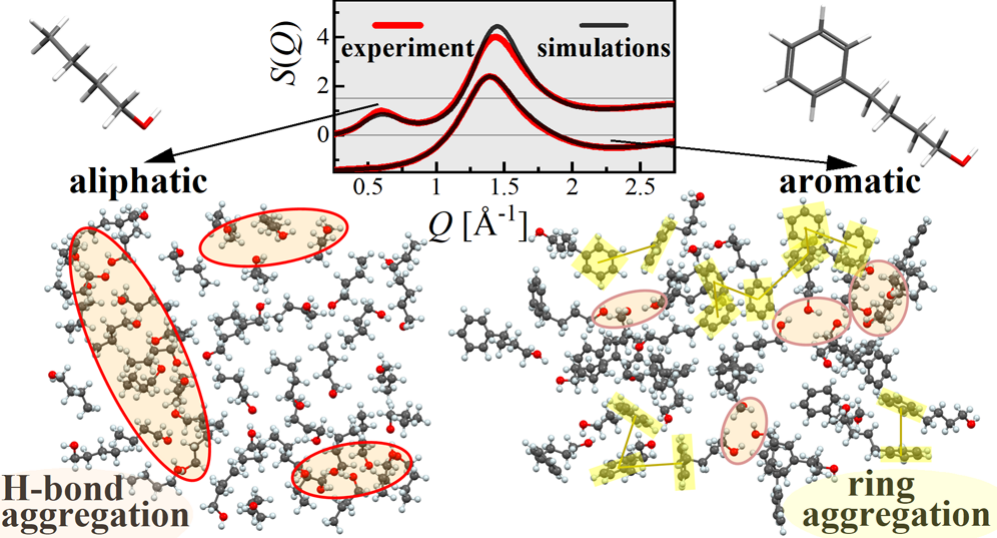

Wide-angle X-ray scattering patterns were recorded for a series of aliphatic butanol isomers (*n*-, *iso*-, *sec*-, *tert*-butanol) and their phenyl derivatives (4-phenyl-1-butanol, 2-methyl-3-phenyl-1-propanol, 4-phenyl-2-butanol, and 2-methyl-1-phenyl-2-propanol, respectively) to determine their atomic-scale structure with particular emphasis on the formation of supramolecular clusters. In addition, molecular dynamics simulations were carried out and yielded good agreement with experimental data. The combination of experimental and theoretical results allowed clarification of the origin of the pre-peak appearing at low scattering angles for the aliphatic butanols and its absence for their phenyl counterparts. It was demonstrated that the location of the hydroxyl group in the molecule of alkyl butanol, its geometry, and rigidity determine the morphology of the supramolecular clusters, while the addition of the aromatic moiety causes more disordered organization of molecules. The phenyl group significantly decreases the number of hydrogen bonds and size of the supramolecular clusters formed via the O–H···O scheme. The lower association ability of phenyl alcohols via H-bonds is additionally attenuated by the appearance of competing π–π configurations evidenced by the structural models.

## 1 Introduction

In ordinary “nonassociating” liquids, the intermolecular structure is isotropic and the structural correlations between molecules are usually lost beyond the second-neighbor shell. However, in many liquids, specific interactions, which include, e.g., hydrogen bonding, hydrophobic relations, π–π stacking, van der Waals, or dipole–dipole forces, can induce spontaneous self-assembly of molecules into aggregates without any external trigger. One example is monohydroxy alcohols, which form supramolecular clusters through hydrogen bonds (HBs) and exhibit a much longer correlation length than ordinary liquids. Despite the growing number of studies for alcohols in binary mixtures, at various interfaces, or in nanoconfinement,^1−4^ there is still a lack of thorough understanding of their association ability in neat bulk forms. One class of alcohols that is largely unexamined in this regard is phenyl alcohols.

Recent years have seen a huge development in molecular dynamics (MD) as a technique to simulate the dynamics and structure of alcohols. Several studies were conducted comparing with a good agreement experimental and simulated total scattering (diffraction) data, e.g., for water–ethanol mixtures,^5^*n*-pentanol and pentanal mixtures,^6^ and neat linear alcohols.^7,8^ To the best of our knowledge, there are no such studies for phenyl alcohols such as phenyl derivatives of butanols. In contrast, the structure of aliphatic butanols has been widely reported in the literature. It was shown experimentally or/and using molecular dynamics simulations that *n*- and *sec*-butanols create chainlike H-bonded clusters,^9−11^ while *tert*-butanol has a tendency to create cyclic structures.^9,10,12,13^ It was also postulated that the steric effect of *tert*-butanol’s globular shape is an obstacle for creating larger supramolecular clusters.^14^

In fact, the discussions on the steric hindrance effect of molecular shape, the impact of the alkyl chain length, and the position of the OH group on the supramolecular structure in various alcohols are abundant in the literature.^15−19^ However, the information on the associating phenomena of alcohols with the steric hindrance in the form of the attached phenyl group is based practically only on the dielectric and infrared spectroscopy studies and many contradictions have arisen on this topic.^20−28^ First, Kalinovskaya et al.^27^ and Johari et al.^28^ postulated that the phenyl group in 1-phenyl-1-propanol reduces the extent of intermolecular H-bonding as the Debye-type relaxation process vanishes. Subsequently, Böhmer et al.^26^ concluded that the aromatic ring only affects the supramolecular architecture of phenyl-propanols, while the structure formation through HBs is not generally suppressed by the increased steric hindrance. Thus, the two above hypotheses were mutually exclusive. Our recent results based on the combination of calorimetric, dielectric, infrared, and diffraction studies suggested that HBs are effectively formed in phenyl alcohols, irrespectively of the steric effect of the aromatic ring. The major factors deciding their degree of association and morphology are the intramolecular architecture and the location of the OH group in relation to the carbon skeleton.^20,22^ In turn, in another paper,^24^ we demonstrated that the phenyl ring exerts a strong effect on the self-organization of 1-phenyl alcohols’ molecules, leading to a significant decline in the size and concentration of H-bonded clusters. Furthermore, it was postulated that besides playing the role of steric hindrance, the bulky aromatic ring acts as a source of additional π···π/OH···π interactions affecting the supramolecular organization. However, this hypothesis needs a strong verification.

With regard to the gap in understanding of the supramolecular assembly and structure of phenyl alcohols, in the current study, we focus on a series of structural alkyl butanol isomers and their phenyl counterparts. To get a deeper insight into the influence of the molecular geometry, location of the hydroxyl group, and, most of all, the presence of the steric hindrance posed by the phenyl moiety on the association of molecules, molecular dynamics simulations were employed. The optimized models of the studied alcohols show very good compliance with the experimental total X-ray diffraction data in real and reciprocal spaces and, therefore, they can be used to interpret the characteristic features of their supramolecular structure.

## 2 Experimental Section

### 2.1 Materials

**Figure 1:**
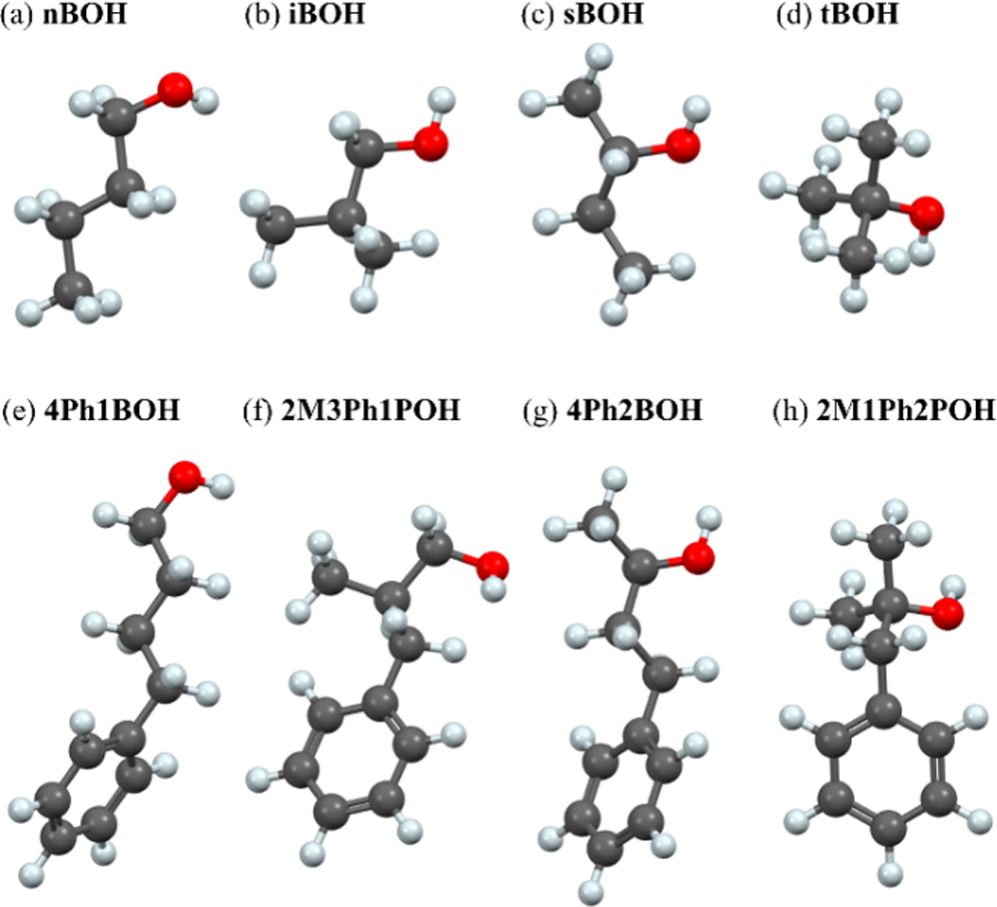
Models of molecules of the investigated alcohols: *n*-butanol (a), isobutanol (b), *sec*-butanol (c), *tert*-butanol (d), 4-phenyl-1-butanol (e), 2-methyl-3-phenyl-1-propanol (f), 4-phenyl-2-butanol (g), and 2-methyl-1-phenyl-2-propanol (h). The abbreviated names used in the article are given above the molecules. Carbon atoms are marked in dark gray, oxygen in red, and hydrogen in light gray.

Aliphatic butanols with the chemical formula C_4_H_10_O: *n*-butanol (nBOH), isobutanol (iBOH), *sec*-butanol (sBOH), and *tert*-butanol (tBOH), and their phenyl derivatives with the chemical formula C_10_H_14_O: 4-phenyl-1-butanol (4Ph1BOH), 2-methyl-3-phenyl-1-propanol (2M3Ph1POH), 4-phenyl-2-butanol (4Ph2BOH), and 2-methyl-1-phenyl-2-propanol (2M1Ph2POH) with purity of at least 97% were purchased from Sigma-Aldrich. For simplicity, we will refer to these phenyl derivatives of butanols as phenyl butanols later in the text. The models of the chemical structure of all studied alcohols are presented in Figure 1.

### 2.2 X-ray Diffraction Measurements

Wide-angle X-ray diffraction (XRD) measurements were carried out on a Rigaku-Denki D/MAX RAPID II-R diffractometer equipped with a rotating Ag anode, an incident beam (002) graphite monochromator, and a two-dimensional image plate detector, operating in the Debye–Sherrer geometry. Samples were measured in capillaries at around 293 K, except for 2-methyl-1-phenyl-2-propanol, which, due to crystallization, was measured at a higher temperature around 297 K. The two-dimensional XRD patterns were transformed to the one-dimensional functions of the scattering intensity versus the scattering vector *Q* = 4π sin θ/λ, where 2θ is the scattering angle and λ = 0.5608 Å is the wavelength. The maximum value of *Q* in the experiment, *Q*_max_, was 20 Å^–1^. In the next step, the total coherently scattered intensity *I*(*Q*), corrected by background, absorption, polarization, and Compton effects and normalized to electron units by the high-angle method^29^ using in-house software, was converted to the scattering factor *S*(*Q*) using the following formula
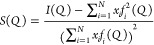
1where *x*_*i*_ is the fraction and *f*_*i*_(*Q*) is the atomic scattering factor of the *i*-th atomic species, respectively, and *N* is the number of atomic species in the sample.

### 2.3 Computational Section

Molecular dynamics simulations were carried out using GROMACS package (version 2020).^30−32^ The calculations were performed at the NVT ensemble (constant volume and temperature), at temperature 297 K for 2-methyl-1-phenyl-2-propanol and at 293 K for the rest of the compounds to represent the laboratory conditions. Each starting simulation box contained 2000 randomly distributed molecules. The size of the cubic box was estimated based on the density of a compound at the given temperature and molar masses of molecules. The values of these parameters in Table SI1 as well as details of the simulations are presented in the Supporting Information. The topology files were created in the Antechamber module (AmberTool21)^33^ with interactions described by the general AMBER force field (GAFF).^34^ The trajectories of the final 100 configurations were collected for further analysis of the systems. Longer simulation time and larger box size were also tested and gave similar results, see Supporting Information, Figures SI6-SI10.

The gmx_rdf, gmx_hbond, gmx_clustsize, and gmx_angle programs in the GROMACS package were used to calculate the partial radial distribution functions of atoms and the radial distribution functions of the center of molecules as well as to analyze the properties of supramolecular clusters, hydrogen bonds, and intramolecular structure. TRAVIS software^35−37^ was used to calculate the partial *S*_*ij*_(*Q*) and total *S*(*Q*) structure factors from the partial radial distribution functions *g*_*ij*_(*r*) as follows
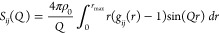
2where the indices *i* and *j* run over *N* different atom types, ρ_0_ is the number density, *r* is the interatomic distance, and *r*_max_ is the maximum sampled distance in the radial distribution function (equal to the box length). Partial structure factors obtained from simulations were multiplied by the weighting factors to get the weighted partial structure factors *S*_*ij*_^′^ (*Q*)
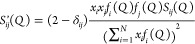
3

where δ_*ij*_ is the Kronecker delta, which sums to the total structure factor *S*(*Q*)
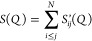
4and can be directly compared with the experimental *S*(*Q*).

## 3 Results and Discussion

### 3.1 Total Structure Factors and Pair Distribution Functions from Experiment and Simulations

**Figure 2:**
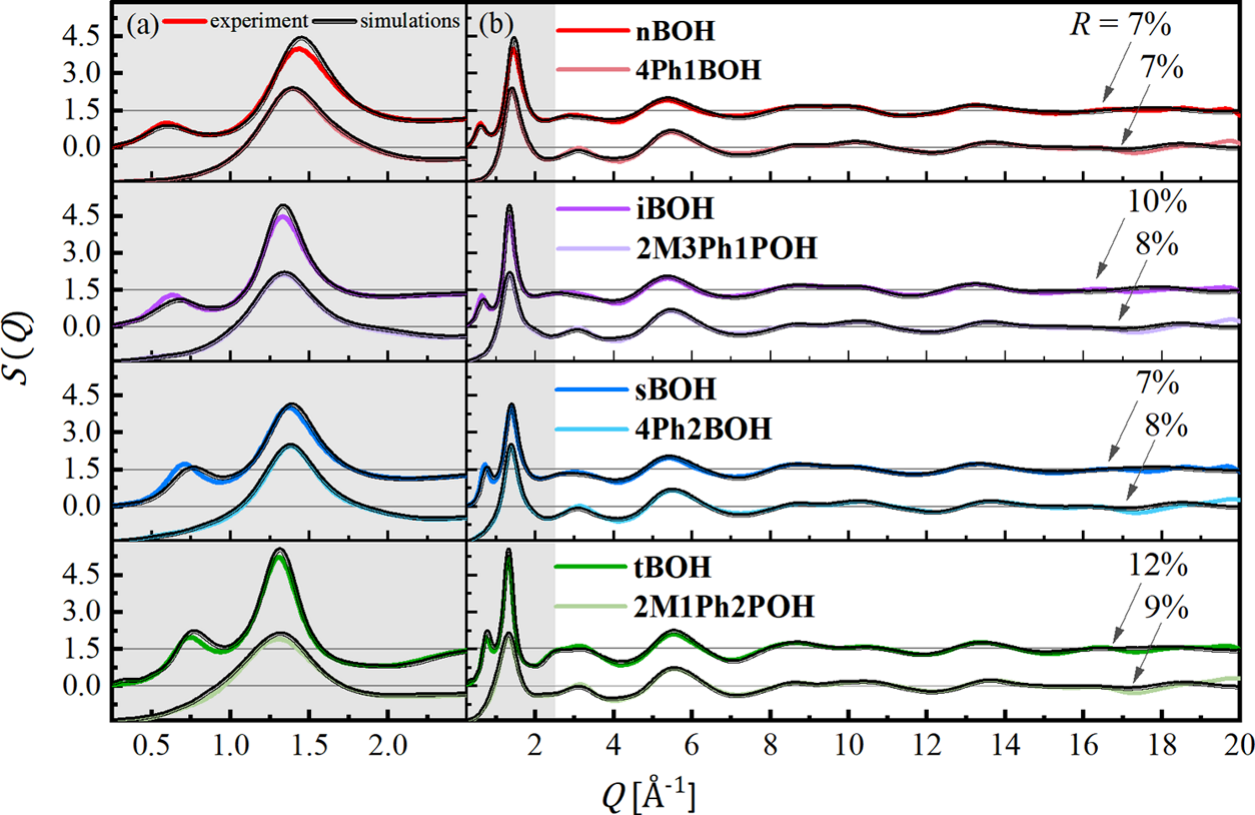
Experimental (colored lines) and simulated (black lines) total structure factors *S*(*Q*) of investigated butanols in the pre-peak and main-peak region (a) and in the whole measured range of the scattering vector *Q* (b). The curves for alkyl butanols are upshifted with the value of 1.5 with respect to their phenyl counterparts. The values of discrepancy factors *R* are given on the right part of the graph.

The experimental structure factors of the different alcohols are compared in Figure 2. In the case of a low-*Q* range (Figure 2a), a dominant main peak (MP) around *Q*_MP_ ≈ 1.3–1.4 Å^–1^ as well as a pre-peak (PP) around *Q*_PP_ ≈ 0.6–0.7 Å^–1^ are observed for each ordinary butanol. However, the positions of both peaks systematically vary for the different isomers. The MP shifts to a higher *Q* value and decreases in intensity with decreasing branching of the molecule, from globular tBOH, through less branched iBOH and sBOH, to linear nBOH. The position of a diffraction peak in reciprocal space can be interpreted in real space through the relation *d* = 2π/*Q*. For dense liquids, the *Q*_MP_ position fingerprints an average particle–particle distance.^8^ The fact that the butanol isomers exhibit different *Q*_MP_ simply reflects the different sizes and geometry of the molecules and their packing ability.  for tBOH, while  for nBOH. Thus, nBOH molecules are more densely packed than tBOH molecules at the same thermodynamic conditions applied, i.e., room temperature and ambient pressure. One may observe similar behavior for phenyl derivatives, i.e., the position of the MP shifts toward a higher *Q* with decreasing branching of the molecules. However, the MPs of phenyl counterparts are visibly wider and have a lower intensity than those of ordinary butanols.

The principal difference in the structure factors between the aliphatic butanols and their aromatic counterparts is the presence of the scattering PP in the low-*Q* region for the former group and its absence for the latter class of alcohols. The origin of the PP in the structure factor of alcohols has been generally attributed to the self-assembly of molecules in aggregates via HBs.^38−40^ It was shown that PP in the total structure factor of neat alcohols becomes the major peak in the partial structure factors involving H-bonding sites, i.e., O–O, H–H, and O–H.^12^ The PP’s position is related to the repeating distance between the hydroxyl head groups in the aggregates and, therefore, to the size of the H-bonded clusters. However, the architecture of the aggregates strongly influences both the PP’s position and amplitude.^39^ In fact, the total PP’s amplitude depends on how different partial atom–atom contributions sum up or cancel out. Therefore, based on solely experimental total *S*(*Q*), it is not possible to assign unambiguously the observed diffraction peaks to specific interatomic correlations. From our previous studies using infrared and dielectric spectroscopy, it was established that the phenyl moiety affects only slightly the degree of association and does not influence the strength of HBs in the phenyl butanols compared to their alkyl counterparts.^20^ Moreover, phenyl butanols were characterized by similar values of the Kirkwood factor, which measures the long-distance correlations between the dipole moments of molecules, to those determined for their aliphatic analogues. In this context, to explain the lack of the PPs in the structure factors of the studied phenyl butanols, in the further part of the paper, the partial atomic contributions *S*_*ij*_^′^(*Q*) to the total *S*(*Q*) were examined based on the optimized models.

A look at the comparison of the experimental and model-based structure factors in Figure 2 allows noticing that the data derived from the MD simulations behave similarly to the experimental ones. The simulation results reproduce the presence of the PP for alkyl butanols and its lack for their phenyl derivatives. Since the low-*Q* region is assigned mainly to the medium-range intermolecular correlations, the good agreement between the model-based and the experimental data in this region is essential for the appropriate characterization of the supramolecular structure and quantifying the effect of the phenyl group on the molecular assembly. The positions, widths, and amplitudes of the PP and MP in the simulated *S*(*Q*) fit well the data derived from the XRD experiment for each alcohol. Also, the oscillations arising mainly due to the intramolecular correlations, observed for higher *Q* values in Figure 2b, are well reproduced by the functions derived from the MD computations. The agreement between the total model-based, *S*_M_(*Q*), and experimental, *S*_EX_(*Q*), structure factors was quantified using the discrepancy factor, , where index *k* runs over the whole *Q* range with a step of 0.01 Å^–1^. The values of *R*, given in Figure 2, are below 10% for almost all alcohols. Thus, the obtained MD models provide a reasonable description of the entire molecular organization of the studied systems at different length scales.

The intermolecular correlations can be also probed by the oscillations of the total pair distribution functions, which are presented in Figure SI1 in the Supporting Information. The functions show that the aliphatic butanols are characterized by longer-range correlations, extending up to around 30 Å. In turn, for phenyl butanols, there are no oscillations beyond around 20 Å. It indicates suppression of the intermolecular order due to the presence of an aromatic ring. In an attempt to understand these observations more deeply, as a trace of the supramolecular aggregation, the data derived from the MD models were further analyzed.

### 3.2 Model-Based Structural Correlations

**Figure 3:**
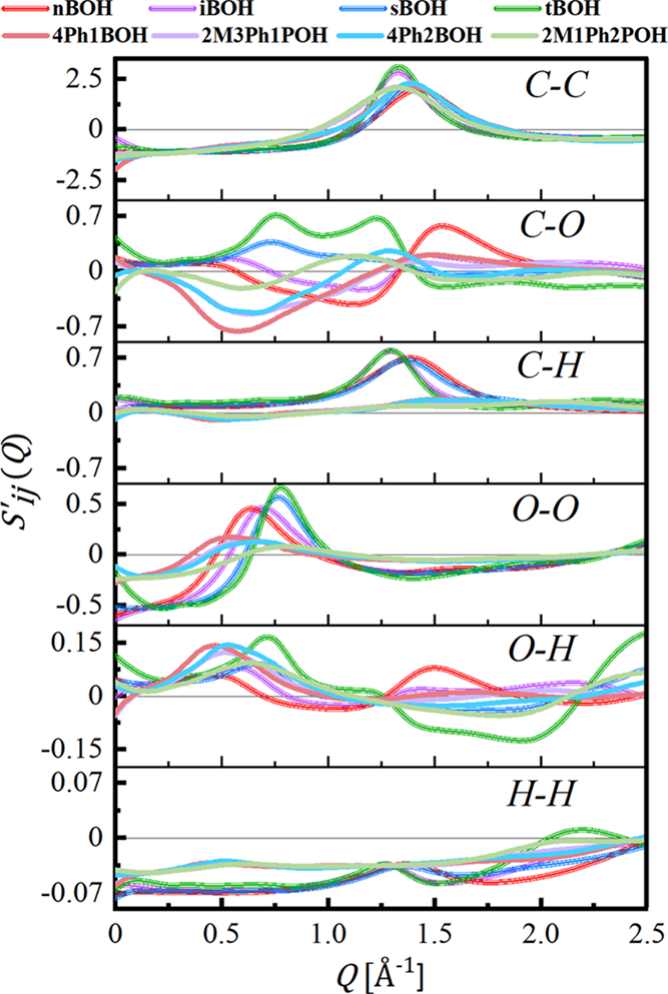
Partial structure factors *S*_*ij*_^′^(*Q*) calculated based on the optimized molecular dynamics models of the investigated butanols.

The calculated partial scattering contributions *S*_*ij*_^′^(*Q*) to the total scattering of the modeled systems, according to eq 4, are depicted in Figure 3. The analysis of their positive and negative parts reveals the origin of the pre-peak in the total *S*(*Q*) functions for aliphatic butanols and its lack for phenyl derivatives of the butanols. The strongest positive contribution to the PP region comes from *S’*_OO_(*Q*) correlations, which are the fingerprint of the H-bonding organization. The positive first peak in *S’*_OO_(*Q*) is observed for both aliphatic and phenyl butanols, indicating the existence of a periodicity in the arrangement of oxygen atoms for all alcohols. However, for phenyl derivatives of butanols, the intensity of this peak is significantly lower and its position is shifted toward lower *Q* values compared to the aliphatic counterparts. There is a greater disorder in the organization of oxygen sites and the O–O correlation length is greater for phenyl alcohols. For instance, the correlation length  for nBOH while  for 4Ph1BOH, which is understandable given the different sizes of the molecules. *S’*_OH_(*Q*), also associated largely with structural correlations of HBs, reveals as well a positive bump in the low-*Q* region around 0.5–0.7 Å^–1^. Interestingly, *S’*_CO_(*Q*) is strongly negative in that region for all phenyl butanols. As a result, positive and negative partial contributions to the total scattering cancel out and no pre-peak is observed.

In turn, for aliphatic nBOH, iBOH, sBOH, and tBOH, the higher intensity of the *S’*_CO_(*Q*) as well as strong *S’*_OO_(*Q*) correlations in this range leads, in consequence, to clear PP feature in total *S*(*Q*) around 0.6–0.7 Å^–1^ for aliphatic butanols. It is also worth indicating that there are considerable differences in the partial contributions to total *S*(*Q*) between the different butanol isomers that explain the various amplitude of the PP. Tertiary tBOH alcohol shows the strongest positive *S’*_OO_(*Q*), *S’*_OH_(*Q*), and *S’*_CO_(*Q*) correlations, while for primary nBOH, *S’*_CO_(*Q*) gives negative correlations in the PP region. Consequently, one may observe that the PP in the experimental functions has the highest intensity for tBOH and the lowest intensity for nBOH. Such a characteristic suggests that the cluster structure of the latter system is less pronounced than that of the former. It is also interesting to note that *S’*_CC_(*Q*) contributes mostly to the main peak of the total *S*(*Q*) for all studied butanols.

**Figure 4:**
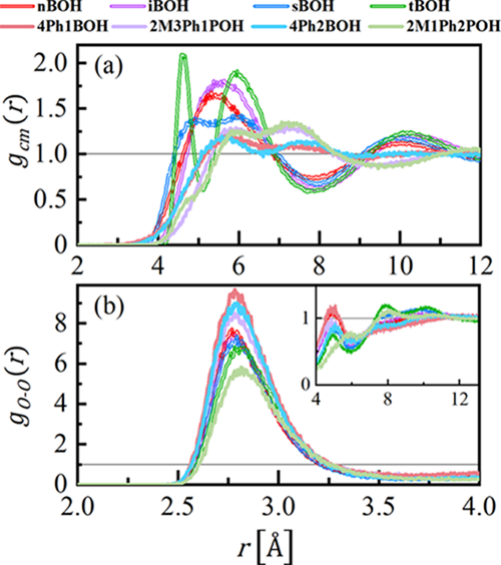
Center of mass *g*_cm_(*r*) (a) and oxygen–oxygen *g*_OO_(*r*) (b) radial distribution functions obtained from the optimized structural models. The inset shows the *g*_OO_(*r*) correlations for longer distances.

In Figure 4, the selected site–site pair distribution functions for all butanols were compared to complement the information given by the structure factors. Figure 4a shows the correlations between mass centers of molecules *g*_cm_(*r*). The first peaks in the *g*_cm_(*r*) function comprise the short-range behavior due to interactions between neighboring molecules. The first distinguishing features of *g*_cm_(*r*) are two pronounced peaks for tBOH, at around 4.6 and 6 Å. They indicate the spatial heterogeneity of the local structure of this alcohol in the near-neighbor range. The first maximum may be identified as a distance between two neighboring molecules aggregated within a cluster, while the second maximum is the distance between the center of masses for the nearest molecules involved in separate clusters. These distances were marked on the structural model presented further in Figure 6c. Similar two-component behavior of the nearest-neighbor distances, but with considerably lower intensity, can be observed for secondary sBOH. For the primary butanols, nBOH and iBOH, the nearest-neighbor intermolecular structure is much more homogeneous with one maximum at around 5.5 Å. For phenyl derivatives of butanols, the correlations between molecules in the nearest-neighbor region are significantly weaker and shifted toward greater distances, as expected, due to the increase in the size of the molecules after attaching the phenyl group. The addition of the phenyl moiety leads also to the heterogeneity in the short-range organization of molecules that can be observed as the appearance of two maxima in *g*_cm_(*r*), at around 5.5 and 7.5 Å. All *g*_cm_(*r*) functions for the different phenyl butanols behave this way and have a related shape that indicates their short-range intermolecular structure is similar.

Figure 4b shows correlations involving oxygen sites *g*_OO_(*r*). Since each of the investigated butanols contains only one oxygen atom involved in the H-bonding per one molecule, the *g*_OO_(*r*) function provides information on the structure of HBs. All other partial *g*_*ij*_(*r*) functions are shown in Figure SI2 in the Supporting Information. The strong first peak in the *g*_OO_(*r*) distribution appears at a distance of around 2.8 Å, which is the generally accepted length for the O–H···O bond,^41^ and witnesses the strong association of oxygen atoms in clusters. The position of the first maxima does not differ significantly between the different butanols; however, it can be noted that in each pair of aliphatic–aromatic butanol, the maximum is shifted slightly toward greater distances for phenyl alcohol. It means that the strength of the HBs is somewhat lowered after the addition of the phenyl group to the molecule. The strongest O–O correlations in the nearest-neighbor range show nBOH, while the weakest show 2M1Ph2POH, which is the most sterically hindered system. The first maxima in the *g*_OO_(*r*) are followed by depleted correlations due to a lower number of the O–O neighbors. It is interesting that all *g*_OO_(*r*) functions show oscillations up to distances around 12 Å (Figure 4b, inset). This observation denotes that the formation of the medium-range order between molecules in the studied butanols takes place with the participation of the O–O correlations resulting from the O–H···O bonds.

### 3.3 H-bonding and Supramolecular Clusters

In the previous section, it was demonstrated that the formation of the medium-range order in the studied butanol alcohols and their phenyl derivatives is due to the intermolecular H-bonding. The molecules form supramolecular clusters via HBs. In general, the analysis of the H-bonding, described in the Supporting Information, indicated that aliphatic butanols form more HBs than their phenyl derivatives, while in the latter group, there are more unbounded molecules. Regardless of the angular conditions imposed on the atoms participating in the H-bonding, the number of all HBs in aliphatic butanol is much higher than in its phenyl analogue, for all isomers (see Figure SI3 in the Supporting Information). The HB distance and angle distributions as well as their average values are shown in Figure SI4 in the Supporting Information. The average HB length is very similar for all alcohols, lying within the limits of 2.81 Å for nBOH and 2.85 Å for 2M1Ph2POH. The average HB angle values are also very close to each other for all systems, around 10°. Moreover, the HB distance and angle distributions for all systems are also similar. It suggests that the H-bonding pattern for all of the studied alcohols is not very different, despite the revealed differences in the number of identified HBs.

Going back to the degree of association of molecules via H-bonds, it is worth mentioning the infrared (IR) spectroscopy results reported in our previous paper.^20^ The IR spectra indicated that at room temperature, aliphatic butanols do not exhibit a signal from unbounded OH moieties while phenyl butanols exhibit a very weak peak associated with vibrations of free OH groups. The numbers of HBs determined here based on the optimized MD models with the broadest angle restriction (H–O–O angle ≤ 90°, see the Supporting Information) best correspond to the results of the previous IR studies. One can see that for this restriction, aliphatic butanols are well associated; however, some molecules are still unbounded. An extension of the angular range for the definition of HB or the complete omission of this restriction could result in a further increase in the degree of association, which would even better match the IR results. Moreover, Gereben & Pusztai^5^ tested different criteria on HBs and stated that sensibly chosen solely distance criteria is sufficient to obtain quantitative results. These findings encouraged us to impose the criterion only on the O–O distance for the analysis of the supramolecular clusters. Thus, the definition ‘cluster’ was used to describe assemblies of H-bonded molecules, which are so close to each other that the distances between intermolecular oxygen atoms do not exceed 3.5 Å and no angle restriction is taken. The term ‘cluster size’ corresponds to the number of molecules in the clusters.

**Figure 5:**
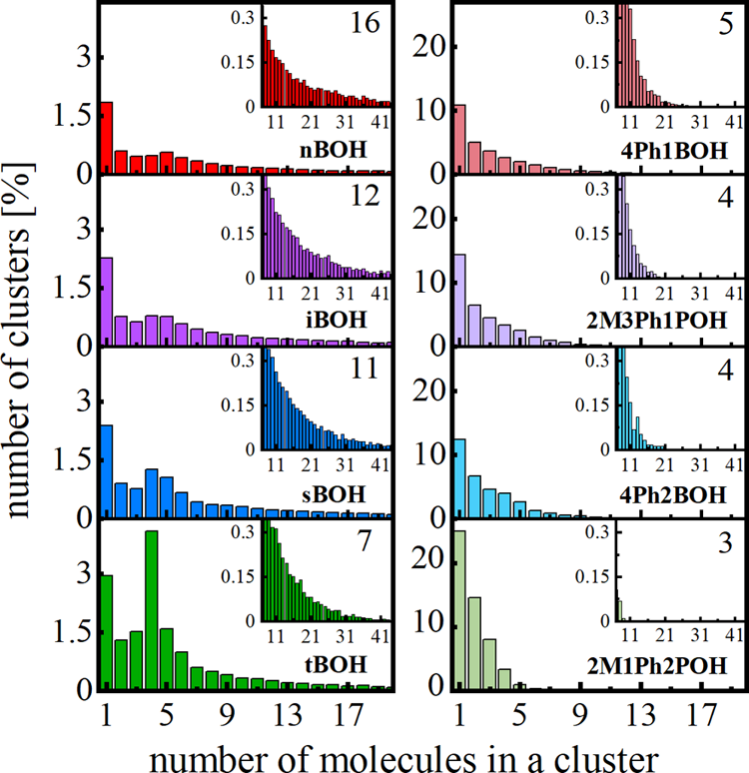
Histograms of the number of clusters as a function of the number of molecules in a cluster. The insets show the distributions in a wider range of cluster sizes. The average number of molecules in the cluster is displayed in the right top corner of each panel.

The calculated histograms of the number of clusters versus the total number of molecules in the clusters are depicted in Figure 5. The first column of these diagrams concerns unassociated molecules. The insets in Figure 5 show the comparison of the histograms for a wider range of cluster sizes. Analyzing the histograms, the following observations can be made:

(1) The number of unassociated molecules increases from around 1.5 to 3% going from primary nBOH, branched primary iBOH, secondary sBOH, and tertiary tBOH. It means that increasing the steric hindrance due to the location of OH group relative to the carbon skeleton and the transformation of the geometry of molecules from linear to globular suppress the clustering ability of these butanols.
(2) For phenyl butanols, this tendency is generally maintained. However, the number of unassociated molecules is much higher (11–24%) for phenyl alcohols compared to their aliphatic counterparts. Thus, it can be argued that the phenyl group is the severe steric hindrance prior to supramolecular clustering.
(3) The width of the cluster size distribution decreases from primary to tertiary alcohols, both for aliphatic and phenyl types. However, for phenyl alcohols, the distributions are much more limited for larger sizes. The narrowest distribution is observed for the most sterically hindered 2M1Ph2POH.
(4) For aliphatic butanols, one can notice a preferential number of molecules in the cluster (local maximum in the distribution): five for nBOH, four to five for iBOH and sBOH, and very pronounced four for tBOH. For phenyl butanols, the number of molecules in the clusters systematically decreases with increasing cluster size and there is no privileged cluster size.

### 3.4 Spatial Models of the Molecular Structure

**Figure 6:**
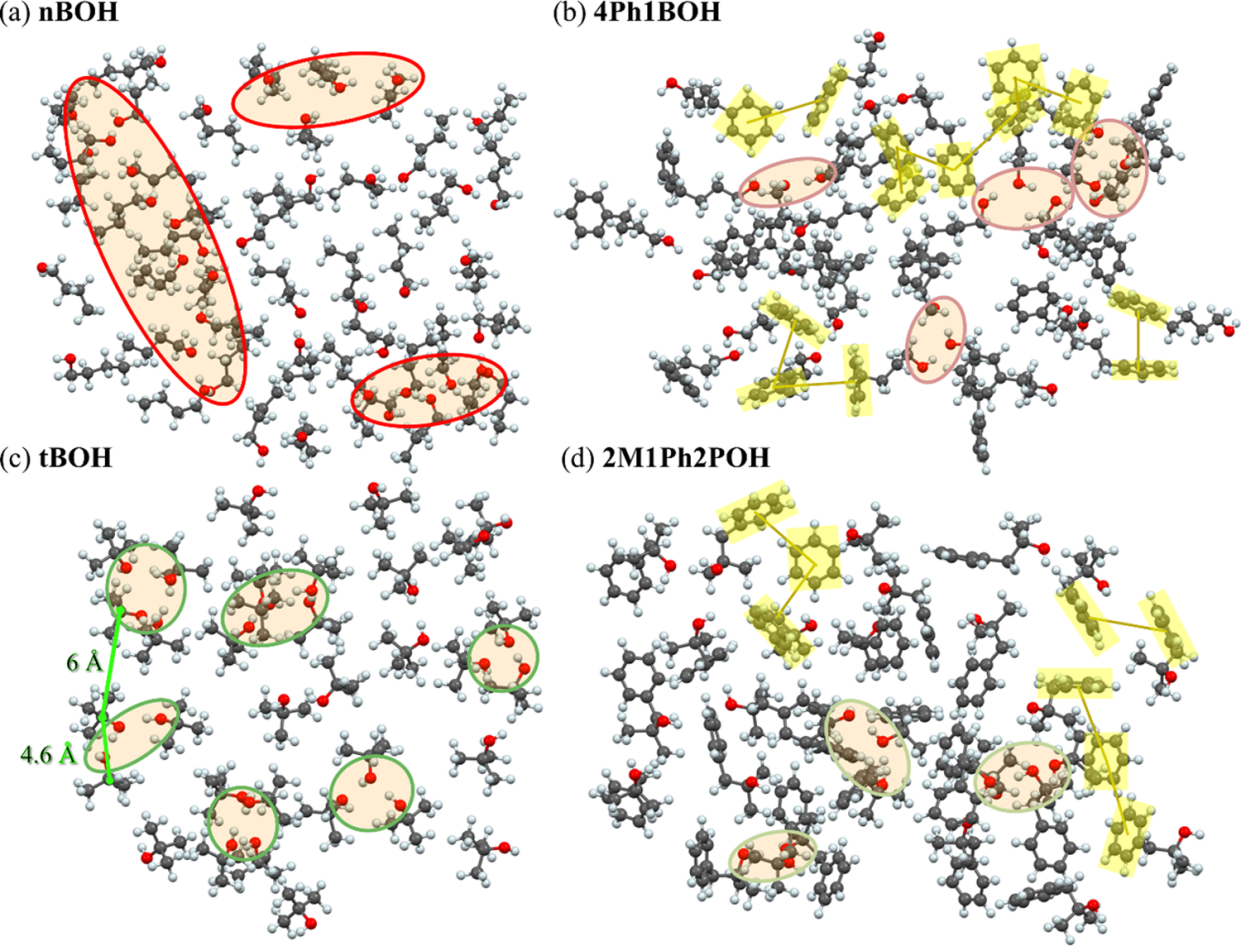
Two-dimensional planes cut from the optimized structural models of nBOH (a), 4Ph1BOH (b), tBOH (c), and 2M1Ph2POH (d). The loops show clusters of H-bonds. The highlighted yellow areas show π–π structural motifs.

The supramolecular organization evidenced by the cluster analysis may be illustrated based on the optimized structural models. To clearly visualize the clustering of the molecules, two-dimensional planes cut from the three-dimensional models were presented for selected butanols and their phenyl derivatives in Figure 6, which summarizes the findings that we intended to report in this paper. The selected model fragments representing compounds from two series, aliphatic and aromatic, illustrate their tendency to associate with molecules. From this picture, one can see that nBOH molecules group in big, longitudinal aggregates where HBs arrange in the chainlike structures (Figure 6a). In comparison, the clusters in tBOH are smaller with rather cyclic geometry (Figure 6c). These findings are consistent with the previous predictions of the architecture of H-bonded clusters for butanol isomers. For example, based on the calculated cluster size distributions, the average number of molecules contained in the aggregates was around 13, 11, 11, and 4 for *n*-, *sec*-, *iso*-, and *tert*-butanol, respectively.^15^ In other computational works,^7,8^ the probability of finding monomers and pentamers in *n*-butanol was the highest. In the case of *tert*-butanol, it was predicted to form either cyclic tetramers^13^ or hexamers.^42^ On the other hand, Figure 6b,d shows the structures of phenyl derivatives of nBOH and tBOH—4Ph1BOH and 2M1Ph2POH, respectively. One may notice a striking difference in the organization of molecules between these two classes of alcohols. For phenyl alcohols, only small supramolecular clusters are formed with a rather chaotic chainlike organization of HBs. It results in a higher degree of spatial disorder than in aliphatic alcohols where more extended networks of HBs direct the arrangement of molecules in the space.

The models shown in Figure 6 verify also the hypothesis of the π···π/OH···π interactions in the phenyl alcohols. In archetypal aromatic benzyl alcohol, several π···π structural motifs for dimers were reported, such as parallel or offset-parallel stacked and perpendicular Y- or T-shaped.^43^ For liquid benzene, the distribution of the nearest-neighbor distances between phenyl ring centers was predicted in the range of 4–7 Å, depending on the offset and angle between the aromatic planes. Taking these values as references for the π···π interactions, it was possible to identify arrangements of molecules in the studied phenyl alcohols meeting these criteria. Such structural motifs were highlighted in the models of phenyl butanols in Figure 6b,d. The π···π configurations were found to occur locally as dimeric of trimeric forms as well as more extended aggregates of several molecules like the one in the right top corner of Figure 6b. In most of these configurations, the aromatic rings are not stacked in parallel face to face but occur in offset and distorted Y- and T-shaped arrangements. Concerning the predicted OH···π bonds, only very sporadic such structural motifs were identified in the models of phenyl butanols. The whole optimized three-dimensional models of all of the studied alcohols are included in the Supporting Information as .pdb files. It should be emphasized that the models do not directly prove the presence of π···π-type interactions but demonstrate the possible configurations of phenyl rings where such interactions would be feasible. Usually, the interactions between aromatic rings or between a functional group and an aromatic ring are not described by general force fields implemented in the classical molecular dynamics simulations. However, here, the GAFF force field was used, which was developed for organic compounds containing phenyl moieties, such as pharmaceuticals, proteins, and nucleic acids.^34^ Therefore, the aromatic ring was distinguishable when calculating the molecular dynamics with the GAFF force field—not at the atomic level but by a different van der Waals bond strength.

It is also worth noting that different molecular conformations were observed in the optimized three-dimensional models of the studied alcohols. The analysis of the molecular conformations, based on the distributions of chosen angles between three atoms in the molecule of each alcohol, is presented in Figure SI5 in the Supporting Information. It was revealed that these distributions for primary and secondary butanols as well as their phenyl derivatives are bimodal. The two components of these distributions correspond to the conformers with bent and linear geometries of molecular skeleton, whereas tertiary butanol and its phenyl derivative occur in only one conformation. That is reasonable taking into account the geometry of these molecules and their rigidity. As predicted, the broadest distributions of molecular conformations are observed for nBOH and its phenyl counterpart, which are very elastic due to the flexible long alkyl tail and the location of OH moiety at its end. Such a molecular structure favors folding of the chain. The flexibility of the molecules and the variability of their conformations facilitate the formation of numerous HBs and big supramolecular clusters, as revealed in the previous section.

## 4 Conclusions

Based on the experimental diffraction studies and molecular dynamics simulations, the supramolecular structure of a series of isomeric butanols and their phenyl derivatives was characterized in detail. The main novel contribution on the topic of the supramolecular clustering of alcohols via H-bonding is the quantitative analysis of the influence of phenyl group attaching to molecules of the butanols on their association ability, the H-bonding pattern, and the structure of the supramolecular clusters. The results allow us to dispel the doubts about whether the steric hindrance in the form of phenyl moiety affects the ability of molecules to link through H-bonds. It was demonstrated that the presence of the phenyl group significantly decreases the number of H-bonds and the size of the supramolecular clusters (the number of molecules aggregated in the clusters), regardless of the location of the OH group in the molecules. The detailed analysis of the atom–atom structure factor contributions exhibited that the presence of the pre-peak in the total structure factor of aliphatic butanols is related to the medium-range order correlations between the OH groups, while the lack of the pre-peak for phenyl butanols is related to weaker O–O correlations as well as the negative contribution coming from the partial C–O correlations, which cancel out.

Furthermore, the analysis of the molecular clustering showed that the distribution of the cluster size changes from broader to narrower while the average number of molecules linked in clusters decreases coming from primary to tertiary butanols. The preference for a specific number of molecules organized in the clusters was displayed, the most prominent for tBOH (4 molecules). In the case of phenyl butanols, clusters were on average 3 times smaller (5–3 molecules) than in aliphatic counterparts (7–16 molecules), with no preferable number of molecules in the cluster size distribution. However, the trend in the H-bonding and clustering properties for phenyl butanols was similar to the series of aliphatic butanols.

Moreover, it was demonstrated that for primary and secondary butanols and their phenyl counterparts, the distribution of the molecular conformations is broad and bimodal. The two components of these distributions correspond to the conformers with bent and linear geometry of molecular skeleton. Thus, the MD simulations showed that the primary and secondary butanols are characterized by higher flexibility of molecular skeletons compared to rigid tertiary butanol. This factor, together with the location of the OH group in the molecule and the presence of the phenyl ring strongly affect the association ability of molecules through H-bonds.

Finally, analysis of the structural models for phenyl butanols allowed us to identify arrangements of molecules where π···π interactions may occur. Such structural motifs may prevent the organization of molecules via H-bonds and enhance the structural disorder. We believe the optimized structural models of the studied butanols and their phenyl derivatives will be helpful for the interpretation of their physical properties. Particularly valuable will be a confrontation of these models with the results of dielectric studies, which so far have yielded many conflicting conclusions about the influence of the phenyl group on the supramolecular association of alcohols and molecular relaxation processes.

## Supporting Information Available

Details of the molecular dynamics simulations and their additional analyses (hydrogen bonds, molecular conformations, results for a longer simulation time and a bigger box size) and the atomic pair distribution function analysis (PDF)
nBOH (PDB)
iBOH (PDB)
sBOH (PDB)
tBOH (PDB)
4Ph1BOH (PDB)
2M3Ph1POH (PDB)
4Ph2BOH (PDB)
2M1Ph2POH (PDB)

## Supplementary Material

jp2c01269_si_001.pdf

jp2c01269_si_002.pdb

jp2c01269_si_003.pdb

jp2c01269_si_004.pdb

jp2c01269_si_005.pdb

jp2c01269_si_006.pdb

jp2c01269_si_007.pdb

jp2c01269_si_008.pdb

jp2c01269_si_009.pdb
