# Supplementary material for: Supramolecular Structure of Phenyl Derivatives of Butanol Isomers
Source: J Phys Chem B. 2022 May 6;126(19):3563–71. doi: 10.1021/acs.jpcb.2c01269 (PMC9125557; doi:10.1021/acs.jpcb.2c01269)
Supplement: Supplementary file 1 — jp2c01269_si_001.pdf [file jp2c01269_si_001.pdf]

Supporting Information for

Supramolecular structure of phenyl derivatives of butanol isomers

Joanna Grelska[1,2,*], *Karolina Jurkiewicz*[1,2,*], *Andrzej Burian*[1,2], *Sebastian Pawlus*[1,2]

[1] A. Chełkowski Institute of Physics, University of Silesia in Katowice,
ul. 75 Pułku Piechoty 1, 41-500 Chorzów, Poland

[2] Silesian Center for Education and Interdisciplinary Research,
ul. 75 Pułku Piechoty 1A, 41-500 Chorzów, Poland

*Correspondence e-mails: joanna.grelska@us.edu.pl, karolina.jurkiewicz@us.edu.pl

1. Calculation of the pair distribution function

The total atomic pair distribution function is defined as follows:

$$G(r) = 4\pi r[\rho(r) - \rho_0] = 4\pi r\rho_0[g(r) - 1]. \qquad \text{[SI1]}$$

The physical meaning of $\rho(r)$ is that $4\pi r^2\rho(r)dr$ determines the number of atoms within the spherical shell of the radius r and the thickness dr, and ρ_0 is the number density. $g(r) = \frac{\rho(r)}{\rho_0}$ gives the actual number density surrounding the reference atom and defines the structural features at the atomic scale. $G(r)$ was determined and calculated using the sine Fourier transform of total $S(Q)$:

$$G(r) = \frac{2}{\pi}\int_0^{Q_{max}} S(Q)\sin(Qr)\frac{\sin(\pi Q/Q_{max})}{\pi Q/Q_{max}}\mathrm{d}Q, \qquad \text{[SI2]}$$

where the last fraction denotes the Lorch modification function reducing effects arising from the finite value of the upper Q limit.

2. Details of the molecular dynamics simulations

Table SI1. Densities and molar masses of the investigated compounds as well as the lengths of the cubic boxes containing 2000 molecules that were taken for the molecular dynamics simulations.

compound name	density [g/cm^3]	molar mass [g/mol]	box length [Å]
**nBOH**	0.81	74.123	67.233
**4Ph1BOH**	0.984	150.22	79.739
**iBOH**	0.802	74.123	67.456
**2M3Ph1POH**	0.9841	150.22	79.736
**sBOH**	0.806	74.123	67.344
**4Ph2BOH**	0.98	150.22	79.847
**tBOH**	0.781	74.123	68.055
**2M1Ph2POH**	0.974	150.22	80.010

It must be stressed out that other force fields, CHARMM27, OPLS-AA, AMBERGS, were also tested with topology files generated by LigParGen server, but the models did not give satisfying agreement with experimental diffraction data. The total charge of studied molecules was equal to zero. The smoothed particle-mesh Ewald (SPME) method treated Coulomb and Van der Waals interactions with cutoff at 20 Å. The intramolecular bond lengths and angles were kept flexible. The time step in simulations was 1 fs. At first, energy minimization was performed by the steepest descent algorithm for 2 ns. Then, equations of motion were integrated for 2 ns using a velocity Verlet algorithm. The temperature was maintained by the Nose-Hoover thermostat, the time constant for coupling was 0.1 ps.

3. The comparison of the experimental and theoretical total atomic pair distribution functions

Apart from the structure factors, the intramolecular and short-range intermolecular correlations can be also probed by the first few peaks of the atomic pair distribution functions presented in Figure SI1a, while longer-range correlations are shown in Figure SI1b. The positions and amplitudes of $G(r)$ peaks derived from simulations are in very good agreement with experimental data in the whole range of r. The model-based functions reconstruct also the differences in the damping of the $G(r)$ peaks for long r between the aliphatic and phenyl butanols. It is clear from Figure SI1b that the former butanols are characterized by longer range intermolecular correlations, extending up to around 30 Å. In turn, for phenyl butanols there are no oscillations beyond around 20 Å. It indicates suppression of the longer-range intermolecular order by the presence of an aromatic ring. In an attempt to understand these observations more deeply, as a trace of the supramolecular aggregation, the data derived from the MD models are further analyzed.

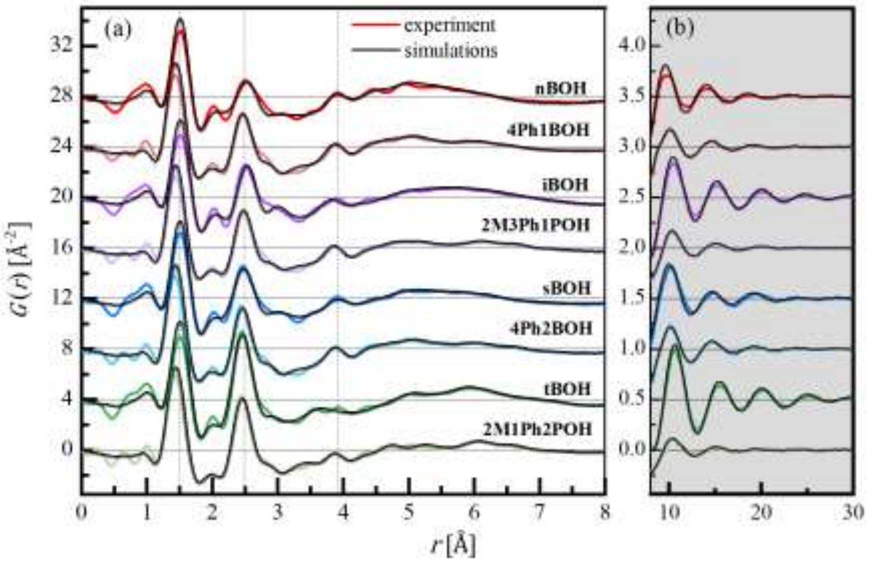

Figure SI1. Experimental (colored lines) and simulated (black lines) total pair distribution functions $G(r)$ of investigated butanols in the region of short (a) and longer (b) distances r. The curves are shifted by 4, starting with 2M1Ph2POH in panel (a). In panel (b), the curves are shifted by 0.5, beginning with 2M1Ph2POH.

4. Partial radial distribution functions

The partial radial distribution functions $g_{ij}(r)$ determined between pairs of atomic species in the system, where the indices i and j run over N different atom types, are shown in Figure SI2. The $g_{OO}(r)$ function is presented in the article in Figure 4.

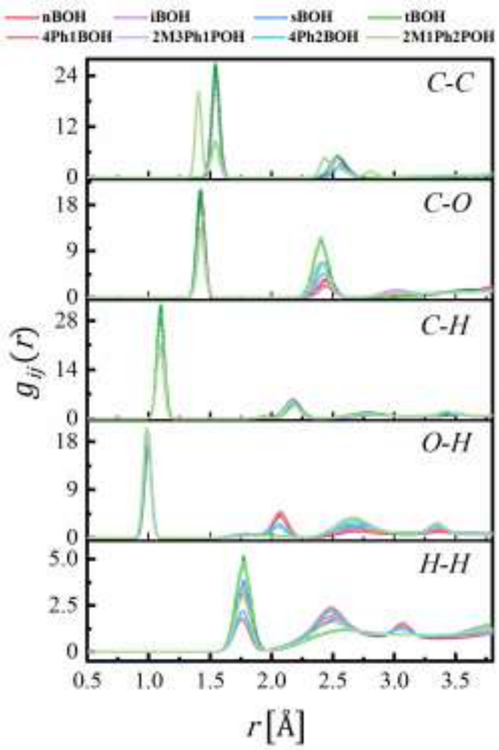

SI2. Partial radial distribution functions $g_{ij}(r)$ obtained from the optimized structural models of butanol isomers and their phenyl derivatives.

5. The analysis of hydrogen bonds and supramolecular clusters

The definition of supramolecular cluster depends crucially on the criteria qualifying how two molecules are linked to each other. Here, H-bonding links are considered. In the case of the studied alcohols, the HBs occur between the electronegative oxygen atom O in one molecule and the positive hydrogen atom H' attached to the oxygen O' from neighboring molecule. This way the linked molecules may form supramolecular clusters of diverse architectures. The number and size of the clusters depend strongly on the connectivity geometrical restrictions

concerning the distance and angle between the atoms forming the O'–H'···O connections, so on the number and geometry of HBs. The maximal distance between O' and O atoms participating in H-bonding is usually taken as a value of the first minimum in the $g_{OO}(r)$ function[1–3]. In many papers the value of 3.5 Å is set[1,4,5] while for the H'–O'–O angle criteria the upper value of 30° or no angle restrictions are the most often applied[1,3,6,7]. In Supporting Information in order to test how the angular criteria are sensitive for estimation of the HBs number, three ranges for H'–O'–O angle were considered: 1) ≤ 30°, 2) ≤ 60° and 3) ≤ 90°. Simultaneously, the upper limit for HB distance was set as the first $g_{OO}(r)$ minimum of 3.5 Å for all butanols (as shown in Figure 3b). The results of the total number of HBs existing in the models and numbers of lone molecules, assuming the above conditions, are presented in Figure. Taking the same conditions, the average HB angles and the HB distances were calculated and are depicted in Figure SI4.

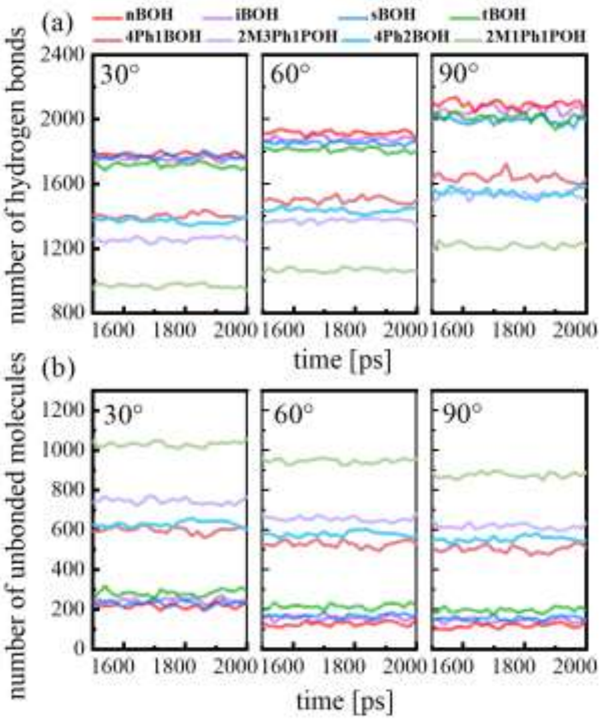

Figure SI3. Fluctuations of the number of all hydrogen bonds (a) and unbounded molecules (b) for last 500 steps of molecular dynamics simulations of the investigated systems calculated for three H'–O'–O angle restrictions: ≤ 30°, ≤ 60°, ≤ 90°, and O'–O distance restriction ≤ 3.5 Å.

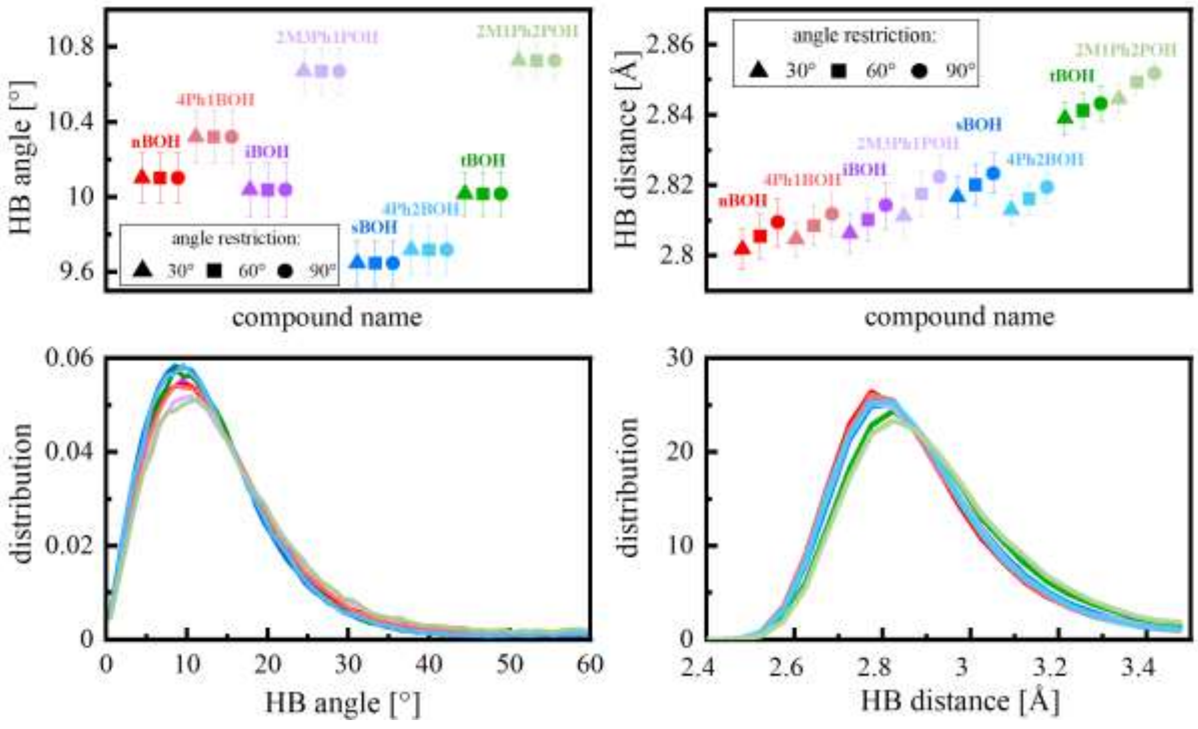

Figure SI4. Average values (top panel) of the distributions (bottom panel) of hydrogen bond (HB) distance and angle, for three H’–O'–O angle restrictions: 1) ≤ 30°, 2) ≤ 60°, and 3) ≤ 90°, and O'–O distance restriction ≤ 3.5 Å. The average values were obtained as central positions of the distributions fitted with Gauss function.

6. Molecular conformations

In the optimized 3-dimensional models of the studied alcohols, different molecular conformations were observed. In order to analyze them, we chose the angle between three atoms in the molecule of each alcohol, which strongly depends on the molecular conformation. Figure SI5 depicts the distributions of these angles for all simulating systems composed of 2000 molecules. In Figure SI5 also the angles corresponding to the maxima of thee angular distributions for aliphatic butanols were graphically marked by orange lines on the models of molecular conformers. For phenyl butanols the same angles were defined as for their aliphatic counterparts. These angles refer to the same atoms in aliphatic alcohols and their corresponding phenyl counterparts, independently of the phenyl ring, while the phenyl

ring is a rigid part of the molecule. The presented distributions disclose that the molecules used for MD simulations resided in one molecular conformation since the simulation box was created as a set of 2000 molecules having the same geometry. After the simulations, in turn, two characteristic types on molecular conformations appeared for primary and secondary butanols as well as their phenyl derivatives. Whereas, tertiary butanol and its phenyl derivative occurred in only one, starting conformation. That is reasonable taking into account the geometry of these molecules and their rigidity. The distributions of the selected intramolecular angles after simulations are much broader than the starting ones as the MD causes fluctuations of the atomic positions. The broadest distributions with two maxima at around 100° and 130° are observed for nBOH and its phenyl counterpart, which are the most elastic due to the flexible long alkyl tail and the location of OH moiety at its end. Such a molecular structure favors folding of the chain. The area under the peak with the maximum for the smaller angle is bigger, suggesting that there are more nBOH molecules with bent geometry of the alkyl tail than with more linear skeleton. Such finding is consistent with previous conformational studies of nBOH by the vibrational spectroscopy and ab initio calculations, which suggested that in the liquid nBOH molecules take diverse conformations[8]. In turn, for iBOH and sBOH, as well for their phenyl counterparts, two distinct conformations appear with a majority of molecules with the smaller angle that refer to starting atom positions. The flexibility of the molecules and the variability of their conformations facilitate the formation of HBs and bigger supramolecular clusters, as revealed in the previous section.

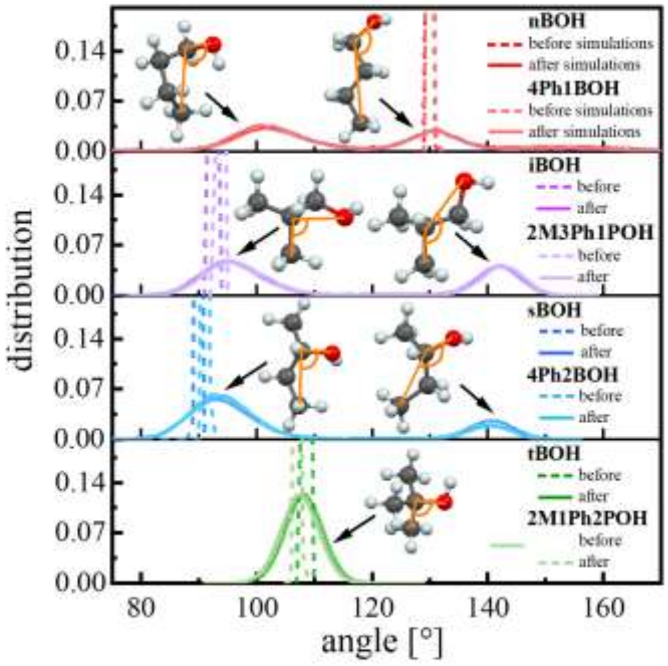

Figure SI5. Distributions of selected intramolecular angles in the studied alcohols before and after MD simulations. The distributions are normalized to 1, but the range of the vertical axis is limited to 0.2 to emphasize the results after MD optimization. The insets show the characteristic molecular conformations for aliphatic butanols with the preferred angles, marked with orange lines, which correspond to the maxima in the distributions.

7. The results of simulations with a longer time and a bigger box size

In order to test the correctness of the chosen simulation time and box size, we performed simulations for the same box size of 2000 molecules but with a longer time of 2 ns as well as for a time of 2 ns but with a bigger box size of 16000 molecules for one of the studied alcohols - isobutanol. The obtained results in the form of: the structure factors, histograms of the number of clusters as a function of the number of molecules, oscillations of the average number of molecules in the clusters with time, distributions of the molecular conformations based on the selected intramolecular angle and oscillations of the angle with the simulation time are presented in Figures SI6-10, respectively. The obtained results demonstrate that the simulation time of 2 ns and the box size of 2000 molecules are enough to obtain stable and statistically-averaged structural properties.

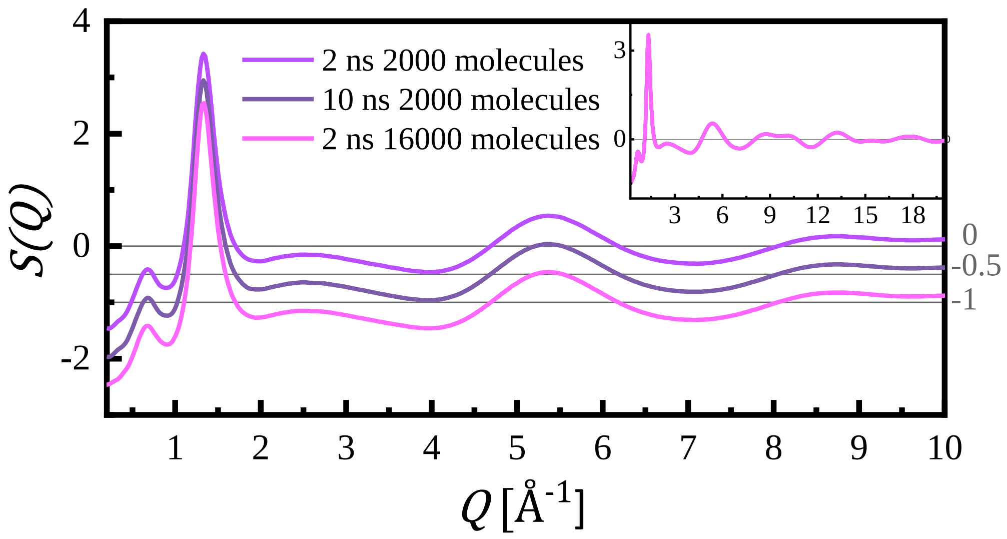

Figure SI6. The comparison of the total structure factors calculated for three models of isobutanol with different parameters: *1*) simulation time of 2 ns and box size of 2000 molecules; *2*) simulation time of 10 ns and box size of 2000 molecules, *3*) simulation time of 2 ns and box size of 16000 molecules. The inset shows that these three structure factors overlap each other.

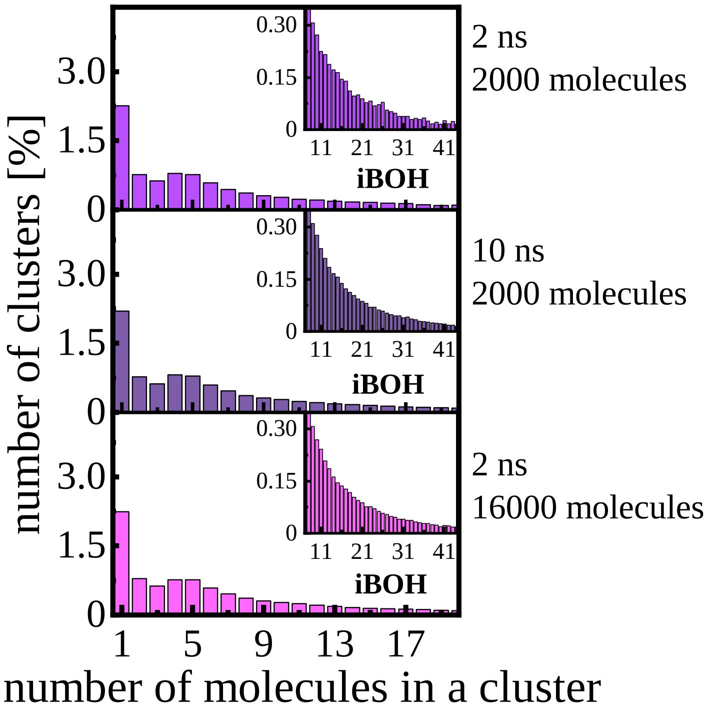

Figure SI7. Histograms of the number of clusters as a function of the number of molecules in the clusters for isobutanol. The results obtained based on three optimized models with: *1*) simulation time of 2 ns and box size of 2000 molecules; *2*) simulation time of 10 ns and box size of 2000 molecules, *3*) simulation time of 2 ns and box size of 16000 molecules.

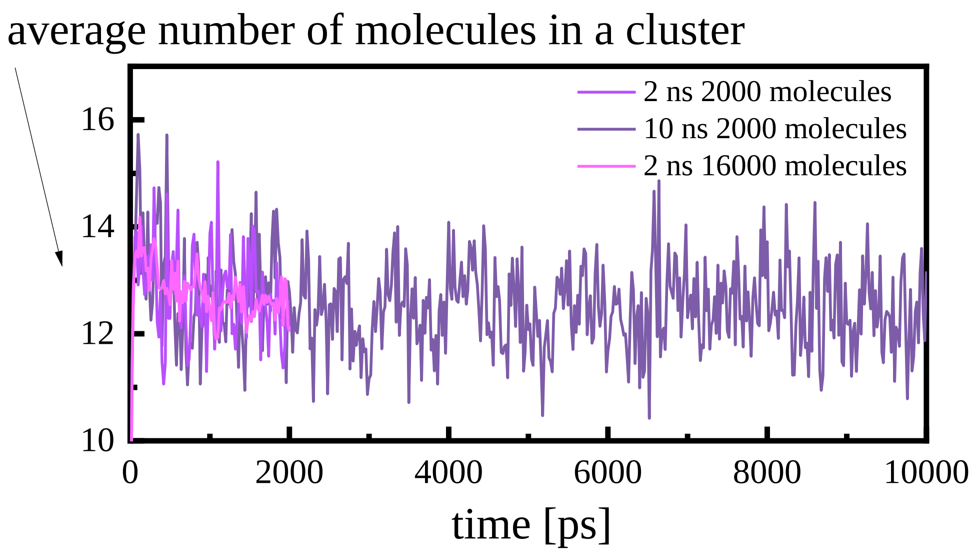

Figure SI8. The oscillations of the average number of molecules in the clusters of isobutanol in time for three models: *1*) simulation time of 2 ns and box size of 2000 molecules; *2*) simulation time of 10 ns and box size of 2000 molecules, *3*) simulation time of 2 ns and box size of 16000 molecules.

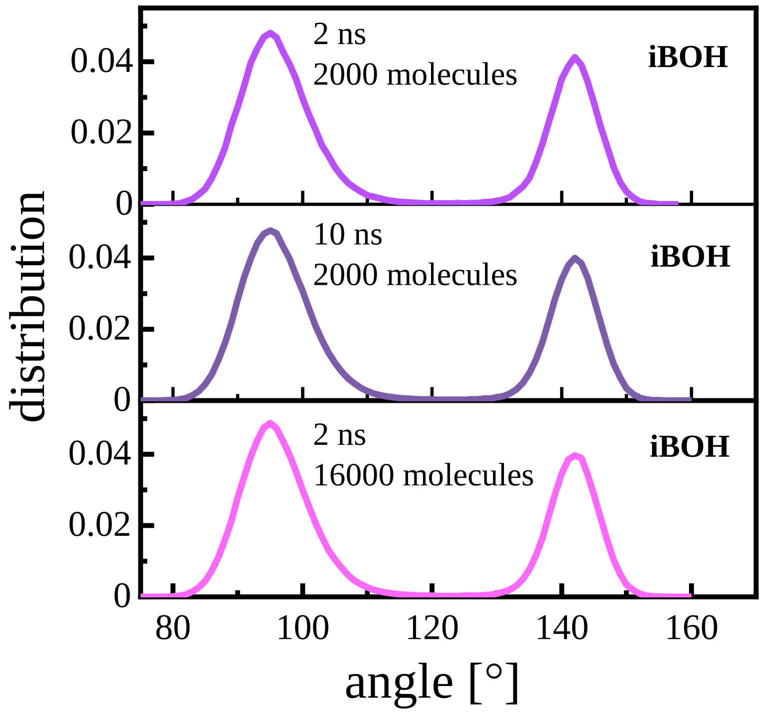

Figure SI9. The comparison of the conformations of isobutanol molecules calculated for three models: *1*) simulation time of 2 ns and box size of 2000 molecules; *2*) simulation time of 10 ns and box size of 2000 molecules, *3*) simulation time of 2 ns and box size of 16000 molecules.

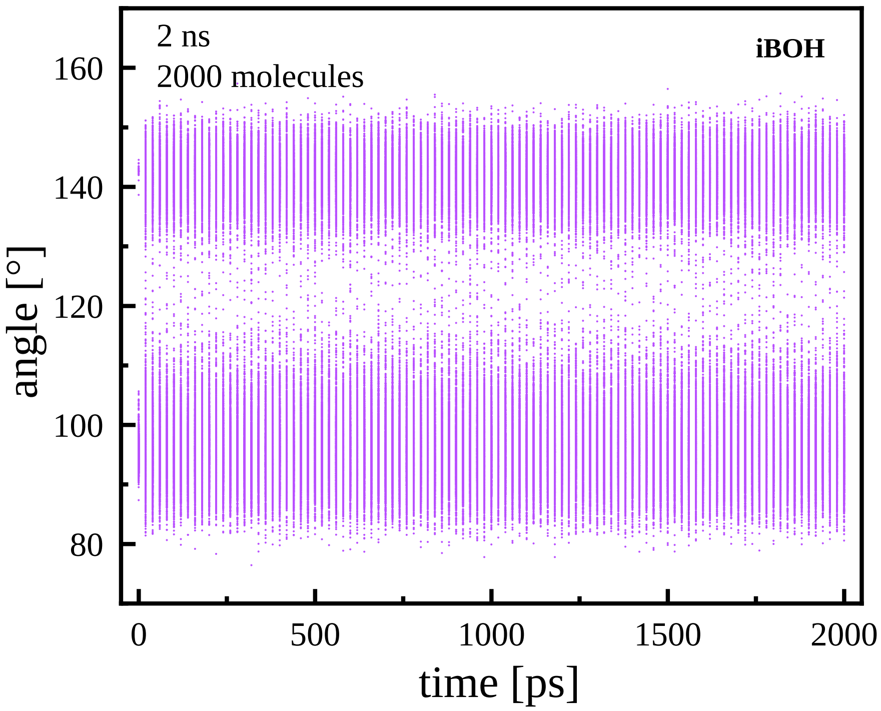

Figure SI10. The distribution of the selected intramolecular angle of isobutanol (indicated in Figure SI5) with the time of the simulations.

References

1. Gereben, O. & Pusztai, L. Hydrogen bond connectivities in water–ethanol mixtures: On the influence of the H-bond definition. *Journal of Molecular Liquids* **220**, 836–841 (2016).
2. Choi, S., Parameswaran, S. & Choi, J.-H. Effects of molecular shape on alcohol aggregation and water hydrogen bond network behavior in butanol isomer solutions. *Phys. Chem. Chem. Phys.* **23**, 12976–12987 (2021).
3. Požar, M. *et al.* Micro-heterogeneity versus clustering in binary mixtures of ethanol with water or alkanes. *Phys. Chem. Chem. Phys.* **18**, 23971–23979 (2016).
4. Geiger, A. & Stanley, H. E. Low-Density 'Patches' in the Hydrogen-Bond Network of Liquid Water: Evidence from Molecular-Dynamics Computer Simulations. *Phys. Rev. Lett.* **49**, 1749–1752 (1982).
5. Oleinikova, A., Brovchenko, I., Geiger, A. & Guillot, B. Percolation of water in aqueous solution and liquid–liquid immiscibility. *The Journal of Chemical Physics* **117**, 3296–3304 (2002).
6. Gómez-Álvarez, P., Romaní, L. & González-Salgado, D. Association effects in pure methanol via Monte Carlo simulations. I. Structure. *The Journal of Chemical Physics* **138**, 044509 (2013).
7. Bolle, J. *et al.* Isomeric effects in structure formation and dielectric dynamics of different octanols. *Phys. Chem. Chem. Phys.* **23**, 24211–24221 (2021).
8. Ohno, K., Yoshida, H., Watanabe, H., Fujita, T. & Matsuura, H. Conformational Study of 1-Butanol by the Combined Use of Vibrational Spectroscopy and ab Initio Molecular Orbital Calculations. *J. Phys. Chem.* **98**, 6924–6930 (1994).
